# Colesevelam for Lenalidomide Associated Diarrhea in Patients with Multiple Myeloma

**DOI:** 10.21203/rs.3.rs-4406606/v1

**Published:** 2024-06-05

**Authors:** Malin Hultcrantz, Hani Hassoun, Neha Korde, Kylee Maclachlan, Sham Mailankody, Dhwani Patel, Urvi Shah, Carlyn Rose Tan, David J. Chung, Oscar Lahoud, Heather Landau, Michael Scordo, Gunjan L Shah, Sergio Giralt, Matthew J Pianko, Miranda Burge, Kelly Barnett, Meghan Salcedo, Julia Caple, Linh Tran, Jenna Blaslov, Tala Shekarkhand, Selena Hamid, David Nemikovski, Andriy Derkach, Oluwatobi Arisa, Cody J Peer, William D. Figg, Saad Z Usmani, Ola Landgren, Alexander M Lesokhin

**Affiliations:** 1 Myeloma Service, Department of Medicine, Memorial Sloan Kettering Cancer Center, New York, NY; 2 Adult Bone Marrow Transplant Service, Department of Medicine, Memorial Sloan Kettering Cancer Center, New York, NY; 3 Division of Hematology/Oncology, Department of Internal Medicine, University of Michigan Medical School, Ann Arbor, MI, USA.; 4 Department of Epidemiology and Biostatistics, Memorial Sloan Kettering Cancer Center, New York, NY; 5 Clinical Pharmacology Program, National Cancer Institute, National Institutes of Health, Bethesda, MD; 6 Myeloma Division, Sylvester Comprehensive Cancer Center, University of Miami, Miami, FL

## Abstract

Lenalidomide maintenance is associated with a significantly improved progression-free in patients with newly diagnosed multiple myeloma. Maintenance with lenalidomide is generally well tolerated; however, lenalidomide associated diarrhea is a common side effect and bile acid malabsorption has been suggested as an underlying mechanism. We conducted a single arm phase 2 trial of colesevelam, a bile acid binder, for lenalidomide-associated diarrhea in multiple myeloma. Patients were treated with colesevelam daily starting at 1250 mg (2 tablets 625 mg) for 12 weeks. The trial included 25 patients, 1 patient with grade 3 diarrhea, 14 with grade 2, and 10 with grade 1 diarrhea. All patients were on treatment with single agent lenalidomide maintenance and no patient progressed during the trial.

Colesevelam treatment was highly effective for treatment of lenalidomide-associated diarrhea; 22 (88%) of the 25 patients responded where 17 patients (68%) had complete resolution of diarrhea, and 5 patients (20%) had improvement by 1 grade of diarrhea. The responses to colesevelam were seen within the first two weeks of treatment. These findings support the conclusion that lenalidomide-associated diarrhea is driven by bile acid malabsorption. Five patients reported mild gastrointestinal side effects including constipation. Importantly, the pharmacokinetics of lenalidomide were not affected by concomitant colesevelam treatment. The stool microbiome composition was not significantly different before and after colesevelam treatment. Patients reported improved diarrhea, fewer gastrointestinal symptoms, and less interference with their daily life after starting colesevelam. In summary, colesevelam was safe and highly effective for treatment of lenalidomide-associated diarrhea in multiple myeloma and does not reduce the clinical effect of lenalidomide.

## Introduction

Lenalidomide is an immunomodulatory drug that is recommended and widely used as part of induction therapy and maintenance for multiple myeloma.^1-4^ Lenalidomide acts via cereblon and induces protein degradation of Ikaros and Ailos eventually leading to myeloma cell death.^5, 6^ Lenalidomide maintenance is associated with a significantly improved progression-free and overall survival as shown in several clinical trials and a meta-analysis.^7-12^ Most patients tolerate lenalidomide well; however, fatigue and diarrhea are common side effects in the maintenance phase. In fact, lenalidomide-associated diarrhea has been reported in up to 20-40% of patients in treated with lenalidomide maintenance.^8, 12, 13^ Diarrhea can have a significant impact on patients’ quality of life and lead to discontinuation of treatment as standard anti-diarrheal medications tend to have a limited effect. In a case series of 12 patients, bile acid malabsorption was reported in lenalidomide-associated diarrhea^14^, and consequently, bile acid binders such as colesevelam have been used in clinical practice. Colesevelam is a bile acid binder approved for the treatment of hypercholesterolemia and to improve glycemic control in adults with type 2 diabetes in combination with diet and exercise.^15^ Furthermore, colesevelam has been found to be beneficial for treatment of diarrhea related to bile acid malabsorption in patients with and without inflammatory bowel disease.^16, 17^

To evaluate the safety and efficacy of colesevelam in multiple myeloma patients in a systematic way, we conducted a phase 2 investigator-initiated trial of colesevelam for patients with lenalidomide-associated diarrhea. An important aspect of the trial was to assess the lenalidomide pharmacokinetics (PK) as lenalidomide has a high bioavailability but the uptake and can be affected by food intake.^18^ Colesevelam is not absorbed systemically^19^ and is assumed to have no effect on lenalidomide PK however this interaction has not been studied. The stool microbiome has been linked to disease outcomes in patients with multiple myeloma and several other hematological malignancies.^20, 21^ We therefore analyzed the stool microbiome composition before and after starting colesevelam to evaluate the potential role in lenalidomide-associated diarrhea. Furthermore, patient reported outcomes regarding gastrointestinal symptoms and their impact on patients’ quality of life were assessed before starting, during and at the end of the treatment with colesevelam.

## Methods

This was an investigator initiated phase 2 single arm open label trial of colesevelam in 25 patients with lenalidomide-associated diarrhea (ClinicalTrials.gov identifier NCT03767257). The study was conducted at Memorial Sloan Kettering Cancer Center, New York between December 2018 and July 2022. The study was approved by the Memorial Sloan Kettering Cancer Center Institutional Review Board and all participants signed informed consent prior to study procedures.

Patients with multiple myeloma who were 18 years or older and on treatment with single agent lenalidomide maintenance with grade 1 or more diarrhea per the Common Terminology Criteria for Adverse Events (CTCAE) v5.0 criteria for at least 4 out of 7 days preceding screening and study inclusion were eligible. Infectious diarrhea was an exclusion criterion and was ruled out (virus, bacteria, parasite and ova) in all patients before enrolling on the trial. Other exclusion criteria were history of bowel obstruction, serum triglyceride levels >300 mg/dL, history of hypertriglyceridemia-induced pancreatitis, and known hypersensitivity to colesevelam. The study design was a Simon 2-stage trial including up to 25 patients. If 5 or more (30%) of the first 16 patients had a clinical response, defined as a decrease in diarrhea by at least 1 grade per CTCAE within the first 4 weeks of treatment, the remaining 9 patients were to be enrolled. Patients were treated with colesevelam daily starting at 1250 mg (2 tablets 625 mg) for a total of 12 weeks. The dose of colesevelam dose could be increased up to a maximum of 6 tablets (3750mg) per day to control the diarrhea. Dose reductions were based on treatment response and adverse events. Patients were instructed to take colesevelam in the morning and lenalidomide in the evening (or at least 4 hours apart). Patient symptoms and CTCAE diarrhea grading were obtained and recorded via clinical trial nurse visits and/or telephone calls at 1, 2, 4, and 12 weeks after starting colesevelam. Adverse events were monitored from the time of signed informed consent to 30 days after end of trial treatment and were reported per CTCAE v5.

### Lenalidomide Pharmacokinetics

Pharmacokinetic (PK) evaluation of lenalidomide was performed before and after colesevelam administration on day 1 and day 8 after starting colesevelam in a subset of 15 patients. Blood samples for lenalidomide PK were collected prior to and 2 hours after the administration of colesevelam. The timing of the lenalidomide dose intake the evening before was recorded. Patients started colesevelam during the lenalidomide cycle (not during the off week) and were assumed to be in a steady state in regards to lenalidomide PK concentration. Lenalidomide has a rapid absorption and follows linear pharmacokinetics with predominantly renal excretion.^18, 22, 23^ Lenalidomide plasma concentrations were measured using a validated high-performance liquid chromatography (HPLC) with tandem mass spectrometric detection (LC-MS/MS) assay, with sensitivity of 5 ng/mL. Patients’ age, renal function, serum creatinine and eGFR per CKD-EPI, as well as weight was recorded and included in the PK model.^23^ Differences in measured pre- or post-dose lenalidomide plasma concentrations were normalized to dose and compared to literature values of single-agent lenalidomide.^18^, ^23^

### Stool microbiome

Stool samples for assessment of the stool microbiome were collected at baseline and at the end of the trial (week 12). Samples were processed within 24 hours of collection and stored at −80°C. DNA was extracted from each fecal sample, and the V4-V5 region of the 16S rRNA gene was PCR-amplified, purified and sequenced on the MiSeq Illumina platform.^21, 24^ The DADA2 pipeline was applied to the sequencing output for quality filtering, removal fo chimeras, and identification of amplicon sequence variants (ASVs).^25^ Taxonomy was assigned via an algorithm utilizing nucleotide BLAST and NCBI refseq.^26^ Analysis was subsequently performed using the phyloseq package in R version 4.2. Taxa known to have enzymatic activities involved bile acid metabolism were identified via literature search. The analysis is done by identifying 62 species from the literature that have known bile salt hydrolase, the bai operon, and other enzymes involved in dehydroxylation, oxidation or epimerization of bile acids.^27-30^ We used the Inverse Simpson to determine α diversity, linear discriminant analysis effect size (LEfSe) method to analyze differences in the composition of stool microbiome in pre- and post-treatment samples.^24, 31^ The Wilcoxon rank sum test was used for comparison between groups.

### Patient reported outcomes

The Patient Reported Outcome CTCAE (PRO-CTCAE) system was developed to measure patient reported outcomes in cancer clinical trials.^32^ Patients were asked to fill out questionnaires to record symptoms and quality of life measures using the PRO-CTCAE gastrointestinal questions. The PRO-CTCAE questions included patient assessment of diarrhea, abdominal pain, constipation appetite, nausea, acid reflux, flatulence, and loss of control of bowel movements, as well as their effect on the patients’ daily life. The PRO-CTCAE questionnaires were performed at baseline, week 1, 2, 4, and 12 (end of trial).

## Results

A total of 25 patients were enrolled and treated on the trial. There were 15 women and 10 men, median age at inclusion was 60 years (Table 1). All patients were on maintenance treatment for multiple myeloma with single agent lenalidomide. Two patients were treated with lenalidomide 5 mg, 19 patients with 10 mg, two patients with 10 mg every other day, and two patients 15 mg of lenalidomide. Twenty-three patients were in first remission while two patients had received more than one line of therapy prior to starting lenalidomide maintenance. The majority of patients had received induction therapy with a three-drug combination including a proteasome inhibitor (carfilzomib or bortezomib), an immunomodulatory drug (lenalidomide), and dexamethasone or a four-drug regimen also including daratumumab. Fourteen patients had undergone high dose melphalan with autologous stem cell transplant prior to starting lenalidomide maintenance. At start of the trial, 18 patients were in a complete remission, 5 patients had a very good partial response and 2 patients had a partial response in regards to the multiple myeloma therapy. All patients maintained their response to multiple myeloma therapy throughout the trial. At baseline, 1 patient had grade 3 diarrhea, 14 had grade 2, and 10 had grade 1 diarrhea, respectively (Table 1). Of the first 16 patients treated in the first stage of the Simon stage 2 design, only 1 did not meet the response criteria and the trial opened to the second stage with the remaining 9 patients.

### Response to colesevelam

**Figure 1.**
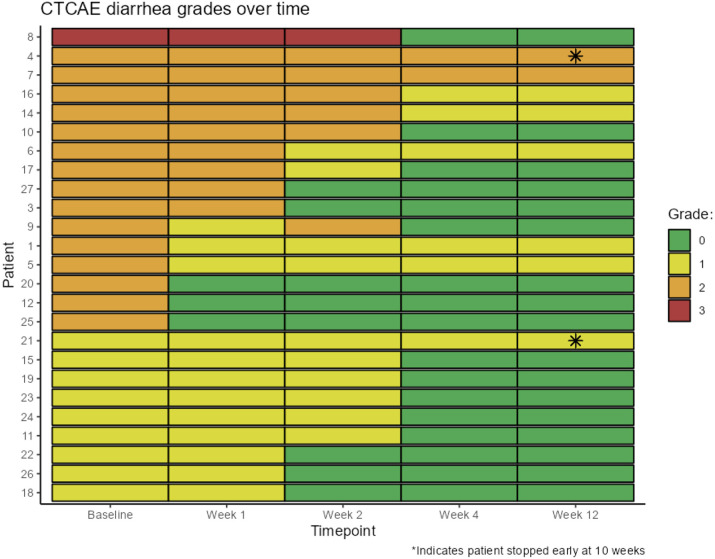
Responses to colesevelam for lenalidomide-associated diarrhea after 12 weeks of colesevelam treatment

Twenty-two patients (88%) responded to treatment with colesevelam with improvement of diarrhea by at least 1 grade per the CTCAE scale. Seventeen patients (68%) had complete resolution of the diarrhea and 5 patients (20%) had improvement by 1 CTCAE grade. Most patients responded to colesevelam therapy within 2 weeks of treatment (Figure 1). Three patients (12%) patients did not respond after colesevelam treatment; 2 had ongoing grade 2 diarrhea and 1 had ongoing grade 1 diarrhea. Thirteen patients continued the starting dose of 1250 mg colesevelam daily, five patients (20%) had a dose reduction to 625 mg colesevelam daily and 3 (12%) patients required dose increase to 1875-2500mg (3-4 pills) per day for control of the diarrhea. Side effects from colesevelam included constipation in 3 patients, flatulence in 1 patient, and acid reflux in 1 patient. Two patients contracted Sars-CoV-2 and one patient had reactivation of shingles while on study treatment with colesevelam (Table 2).

### Pharmacokinetic evaluation of lenalidomide

**Figure 2.**
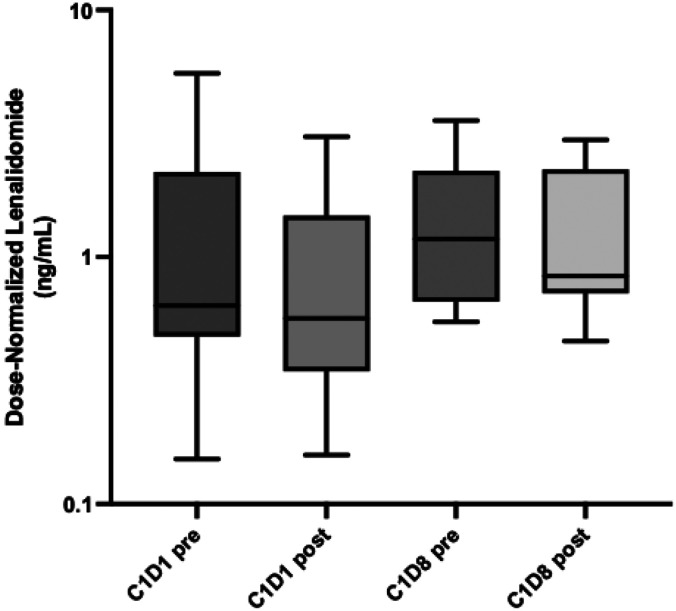
Dose normalized lenalidomide pharmacokinetics before and after starting colesevelam

Lenalidomide PK was measrured on day 1 and day 8 of starting colesevelam. The time since lenalidomide administration ranged between 10-13 hours. There was no significant difference in lenalidomide PK before and after starting colesevelam on day 1 and 8 confirmning that the lenalidomide concentration was not affected by colesevelam (Figure 2). The dose-normalized lenalidomide concentration for pre- and post-C1D1 were 1.23 ng/mL and 0.84 ng/mL, while the pre- and post-C1D8 were 1.58 ng/mL and 1.29 ng/mL, respectively. The range was 0.5-5 ng/mL, which is similar to PK measurement in early lenalidomide PK studies in healthy controls.^18, 23^

### Stool microbiome analysis

**Figure 3.**
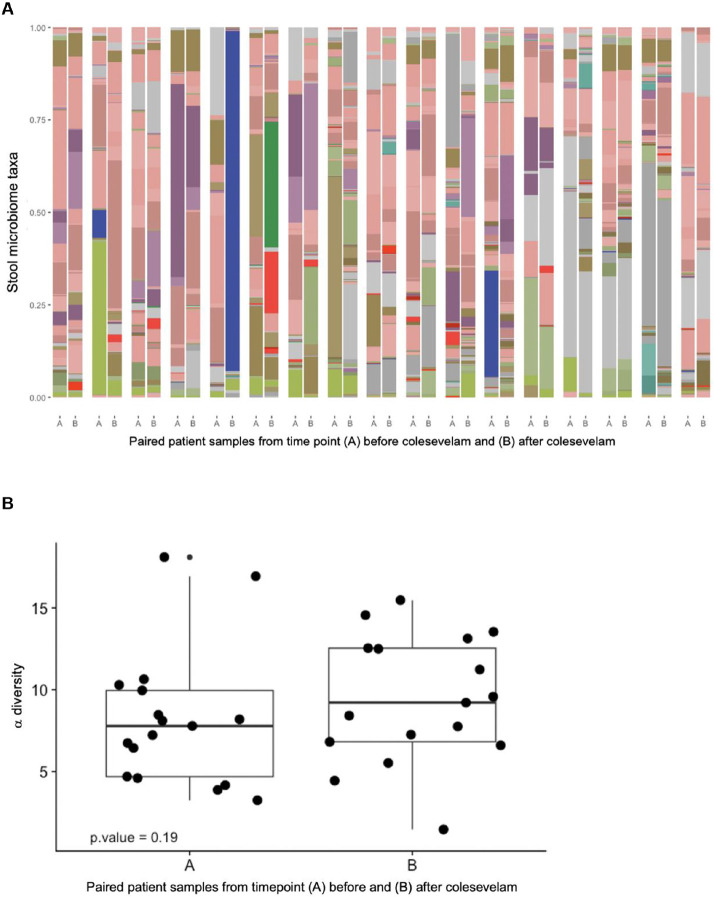
Stool microbiome before and after treatment with colesevelam. (A) Taxa before and after colesevelam. (B) α diversity in pre- and post colesevelam treatment stool samples. (C) Abundance of bile acid enzyme producing bacteria in formed and liquid/soft stool samples.

There were no significant differences in α-diversity or composition of gut bacteria before and after starting colesevelam (Figure 3A). The α-diversity tended to be greater after treatment with colesevelam possibly related to a higher gastrointestinal retention time when bowel habits normalized (Figure 3B). There was numerically increased abundance of bacterial taxa involved in bile acid metabolism in formed stool samples suggestive of increased bile acid metabolism in the lower intestinal tract, although the difference was not significant (Figure 3C).

### Patient reported outcomes

**Figure 4.**
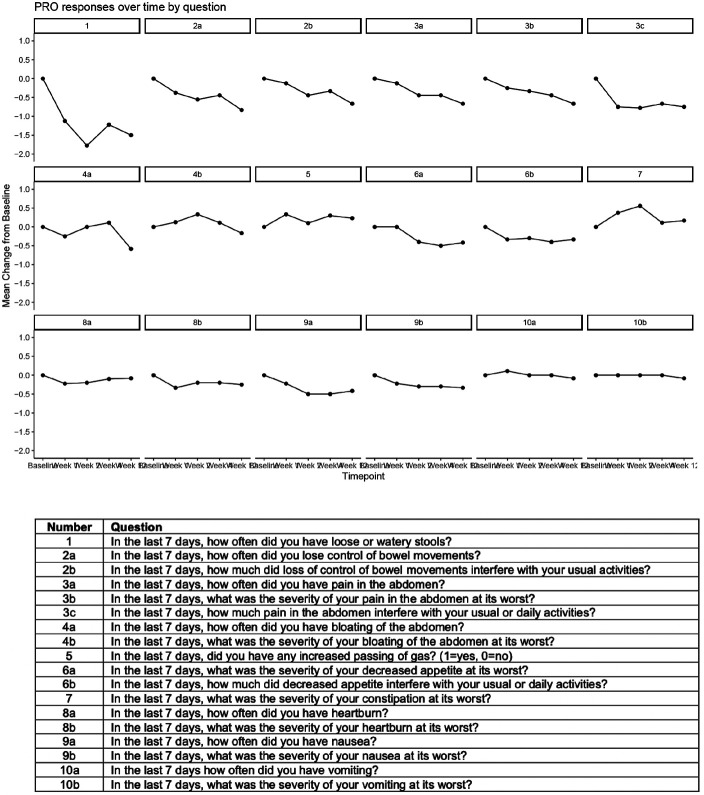
Patient reported outcomes over the 12 weeks of treatment with colesevelam for lenalidomide associated diarrhea

Patient reported outcomes were assessed for gastrointestinal issues and their effects on daily life. In the majority of patients, colesevelam resolved or improved the frequency of watery stool (Figure 4, Supplementary Figure 1). Patients reported better control of their bowel habits, e.g. fewer occasions of loss of control of bowel movements. Patients reported improvements in abdominal pain, heart burn, bloating, and better appetite over the course of the study. Five patients reported increase in constipation, flatulence, heart burn, or nausea after starting colesevelam. Nevertheless, patients graded the effect of the gastrointestinal symptoms on their daily life and activities as less severe after starting colesevelam.

## Discussion

Colesevelam was a highly effective for treatment of lenalidomide-associated diarrhea, with 88% of patients responded with improved symptoms and 68% with complete resolution of diarrhea. Responses were seen shortly, within 1-2 weeks, after starting colesevelam. Importantly, the lenalidomide PK was not altered by colesevelam treatment. Patients reported significant improvements in symptoms and their effect on quality of life after starting colesevelam. Diarrhea associated with lenalidomide is common and can significantly impact the patients’ daily activities and quality of life. This type of diarrhea can cause urgency and abdominal pain and results in patients adjusting their daily activities. It can eventually lead to unnecessary discontinuation of lenalidomide as and standard anti-diarrheal options, such as loperamide, and dose reduction of lenalidomide tend to have limited effects.^14^ So far, the benefits of colesevelam for lenalidomide-associated diarrhea have been reported in one a prospective study of 10 patients as well as an abstract from a retrospective study.^14, 33^ Nevertheless, colesevelam and other bile acid binders have been used off label in clinical practice to treat lenalidomide-associated diarrhea. In our study of patients with grade 1 or more lenalidomide-associated diarrhea, we found a clear benefit and improvement in patients’ symptoms after starting colesevelam. Most patients (68%) had complete resolution of diarrhea and an additional 20% had reduction in the severity of diarrhea.

Patients reported overall improvements in gastrointestinal symptoms and less impact on their daily life after starting colesevelam. There was a reduction in frequency of watery stools and patients reported improvement control of their bowel movements. While on colesevelam, patients reported less abdominal pain, heartburn, nausea, and improved appetite. Side effects from colesevelam were mild and in patients who developed constipation, this could be resolved with dose reduction of colesevelam. Assessing not only response but also PROs is essential as the overall goal was to improve patients’ perceived symptoms and quality of life. Importantly, concomitant treatment with colesevelam did not affect the lenalidomide PK and does thus not reduce the lenalidomide treatment effect. This has not been shown previously and is provides important clinical information for clinicians and patients on the safety of lenalidomide in combination with colesevelam.

There was no significant difference in the diversity or composition of the microbiome before and after colesevelam treatment. There was a trend towards an increased α distribution after colesevelam treatment, this may be an effect of the more normal and longer gastrointestinal transit time after the diarrhea resolved. There was also increased number of bile acid enzyme producing bacteria in liquid stool samples compared to formed stool samples which would be expected if there is more bile availability. One limitation to this analysis was the small sample size and the fact that all stool samples were collected after the onset of diarrhea. Nevertheless, the results indicate that the gut microbiome does not play a significant effect in the etiology of lenalidomide-associated diarrhea.

Strengths of this study include assessing colesevelam for treating lenalidomide-associated diarrhea in a systematic way including several aspects such as outcome, PK data, stool microbiome, and importantly, patient reported outcomes. The study was conducted during the COVID-19 pandemic, due to this, some PRO-CTCAE questionnaires as well as stool samples were not collected as part of the study visits were remote. The PK substudy was a priori planned for 15 patients and included all planned subjects. The reported infections, two COVID-19 and one case of shingles, were assessed as not related to colesevelam treatment but instead to the COVID-19 pandemic and/or the multiple myeloma and lenalidomide treatment.^34^ In summary, colesevelam was highly effective for treatment of lenalidomide-associated diarrhea. Diarrhea is a common side effect during lenalidomide maintenance with significant effect on patients’ quality of life. The high response rate to colesevelam in this and the previous study by Pawlyn et al support bile acid malabsorption as the underlying mechanism for lenalidomide-associated diarrhea. Lenalidomide maintenance prolongs progression free survival and properly managing side effects allows patients to stay on lenalidomide and retain the beneficial effects of maintenance. Treatment with colesevelam can efficiently resolve or reduce the diarrhea while maintaining the clinical benefit of lenalidomide.

## Supplementary Material

Supplement 1

## Data Availability

Data is not publicly available, please contact the corresponding author for questions.
