# Supplementary material for: Colesevelam for Lenalidomide Associated Diarrhea in Patients with Multiple Myeloma
Source: Res Sq. 2024 Jun 5:rs.3.rs-4406606. Preprint. [Version 1] doi: 10.21203/rs.3.rs-4406606/v1 (PMC11177961; doi:10.21203/rs.3.rs-4406606/v1)
Supplement: Supplement 1 [file NIHPPrs4406606v1-supplement-1.pdf]

Supplementary Figure 1. Changes in patient reported outcomes between baseline and end of treatment with colesevelam for lenalidomide associated diarrhea.
